# Risk of Suicide and Self-harm in Kids: The Development of an Algorithm to Identify High-Risk Individuals Within the Children’s Mental Health System

**DOI:** 10.1007/s10578-020-00968-9

**Published:** 2020-02-19

**Authors:** Shannon L. Stewart, Angela Celebre, John P. Hirdes, Jeffrey W. Poss

**Affiliations:** 1 Faculty of Education, Western University, London, ON Canada; 2 Faculty of Applied Health Sciences, University of Waterloo, Waterloo, ON Canada

**Keywords:** Children’s mental health, Suicidal ideation, Self-harm, Suicide risk, interRAI

## Abstract

Suicide is the second leading cause of death in adolescents within Canada. While several risk factors have been found to be associated with increased risk, appropriate decision-support tools are needed to identify children who are at highest risk for suicide and self-harm. The aim of the present study was to develop and validate a methodology for identifying children at heightened risk for self-harm and suicide. Ontario data based on the interRAI Child and Youth Mental Health Screener (ChYMH-S) were analyzed to develop a decision-support algorithm to identify young persons at risk for suicide or self-harm. The algorithm was validated with additional data from 59 agencies and found to be a strong predictor of suicidal ideation and self-harm. The RiSsK algorithm provides a psychometrically sound decision-support tool that may be used to identify children and youth who exhibit signs and symptoms noted to increase the likelihood of suicide and self-harm.

## Introduction

Suicide is a major public health concern that has devastating long-term effects on both families and communities. It has been estimated that suicide and self-harm costs Canadian society approximately $3.3 billion annually in both direct and indirect costs [1]. Notably, suicide is the second leading cause of death among adolescents in Canada [2]. Previous research suggests that effective prevention strategies for child and adolescent suicide should primarily be targeted at reducing suicide risk factors [3]. Furthermore, the risk factors and features of suicidality in youth are different than those found in adults, hence the urgent need to determine potential risk factors as well as develop decision-support tools to identify these young people at greater risk [4].

### Self-harm: Nonsuicidal Self-injury and Suicidal Self-injury

Self-harm is defined as any deliberate and direct act that causes harm to one’s body, encompassing both nonsuicidal self-injury (NSSI) and suicidal self-injury (SSI). NSSI is defined as intentional destruction of one’s body tissue without lethal intent and has a prevalence rate in adolescents of approximately 30–40% in clinical samples and 13–29% in non-clinical samples [5–7]; SSI is deliberate self-directed harm to one’s body with the intent to end one’s life and has a lower prevalence by comparison of 24–33% in clinical samples and 4–8% in non-clinical samples [5, 7, 8]. Adolescence represents a period of heightened risk for the initiation and engagement in self-injurious behaviours, given that the average age of onset is 12–13 years [9].

### Potential Risk Factors for Self-harm, NSSI, and SSI

#### Individual Factors

Psychiatric disorders are highly prevalent in adolescents who self-harm, with depression, anxiety, and substance misuse being the most commonly reported [10–12]. Depression is considered a key factor in the etiology of suicidality in youth, as it is heavily reported in both clinical and community populations [13]. Research has found that major depressive disorder (MDD) is the greatest risk factor for suicide attempts, with higher levels of depressive symptoms being associated with a greater likelihood of engaging in both NSSI and SSI [12, 14].

A prior history of self-injurious behaviour is one of the strongest predictors of future suicidal behaviour, both cross-sectionally and longitudinally [15]. The CASE study found that among those who self-harmed within the year before, more than 50% reported multiple events, suggesting that repetition of self-harm is quite prevalent in adolescents [16]. A review examining the link between NSSI and suicidal behaviour found that across studies, NSSI was consistently a robust predictor of suicidal thoughts and behaviour, above and beyond a number of well-studied factors including depression and family functioning [17–19]. It has also been reported that more frequent engagement of NSSI is predictive of more frequent suicide attempts [17]. Additionally, longitudinal studies have found that a previous suicide attempt increases the risk of a future suicide attempt threefold [20].

Self-harm and suicidal behaviour has also been linked to certain personality and character traits, such as perfectionism and neuroticism [21]. One trait that has become increasingly important in the literature is impulsivity, as it has been reported that 50% of adolescents start thinking about harming themselves less than an hour before performing the act [22]. Impulsivity is known to increase the risk of suicidal behaviour; moreover, adolescents who engage in both NSSI and SSI have significantly higher trait impulsivity compared to those who engage in NSSI only [23]. Self-injury has also been linked to compulsive behaviours, such that having one or more obsessive compulsive symptoms increases the odds of suicidality by 2.4 times [24].

#### Interpersonal Factors

A lack of family and social support is associated with a greater likelihood of engaging in self-harm. Research has found a number of family factors are related to adolescent self-harm, including maladaptive parenting, parental divorce, domestic violence, and child maltreatment [25–27]. Adolescents who have attempted suicide are more likely to report running away from home, stress related to parents, and lack of adult support outside the home [28]. Moreover, a longitudinal study found that even after controlling for depression, low family support predicted future suicide attempts into young adulthood, highlighting the persistent effect of low family support on suicidality [29, 30]. Finally, family discord has been reported as the most common precipitant of completed suicide in adolescents [31].

The present study sought to develop and validate a methodology that could identify children and youth who are at greater risk of suicide and self-harm within the Ontarian children’s mental health system. A validated methodology to identify adults who are at risk for suicide and self-harm (SOS) has previously been developed [32]. Due to the fact that Ontario does not currently have an existing system specifically designed for children and youth, an effort was launched to develop a new decision-support algorithm for identifying those at greatest risk of self-harm in this younger population. Applying the same methodology utilized in the SOS, the Risk of Suicide and Self-Harm in Kids (RiSsK) algorithm was created to assist healthcare providers in determining whether a young person is at heightened risk of self-harm or committing suicide. The aim of this article is to describe the development and validation efforts undertaken as part of the development of the RiSsK algorithm.

## Methods

### Sample

The participants in this study consisted of children and youth who received services from mental health agencies in Ontario. Referrals were made to the agencies through their family physicians, pediatricians, school personnel, parents or other allied professionals. Derivation was conducted on a primary sample using screener records collected between September 1, 2015 and January 31, 2019, a total of 60,414 records on 54,280 unique individuals from 59 organizations. Males made up 49.8% of these observations and the mean age was 11.8 years (SD 3.74, range 4 to 18 years). A validation sample consisted of 2117 records on 2098 unique individuals that were completed in a subsequent time period in the same 59 organizations, between February 1, 2019 and March 5, 2019. Males made up 49.0% of these observations and the mean age was 11.7 years (SD 3.67, range 4 to 18 years). There were no differences in the methods or sources between the derivation and validation samples.

The derivation and validation data came from the implementation of the Child and Youth Mental Health Screener (ChYMH-S) [33], described below. Two additional related sources of data were also used in the post-scale development stage, the Child and Youth Mental Health (ChYMH) [34] and the Child and Youth Mental Health and Developmental Disability (ChYMH-DD) [35], also described below. A sample of 25,104 ChYMH and ChYMH-DD assessments on 13,899 unique individuals was used, completed between September 1, 2015 and January 31, 2019. Males made up 57.0% of these observations and the mean age was 12.1 years (SD 3.51, range 4 to 18 years). The ChYMH, ChYMH-DD, and ChYMH-S have been utilized as the standard of care in mental health agencies across the Province of Ontario. Therefore, the inclusion criteria were children and youth 4–18 years of age who presented at mental health facilities utilizing the interRAI child/youth suite of instruments as standard of care.

### Measures

The ChYMH-S is a brief assessment tool used in assessing, triaging and prioritizing children and youth seeking mental health services [33]. Within the global interRAI Collaborative network, the ChYMH-S was developed to provide seamless screening and support decision-making related to triaging, placement, and service urgency for children and youth with mental health needs. It was also designed to be used in multiple settings, including schools, community programs, as well as inpatient and residential services [33].

The ChYMH-S takes approximately 15–20 minutes to complete, subject to some variability depending on case complexity. The tool comprises approximately 100 items, most of which are binary or ordinal scale measures for a specific time period. Furthermore, it consists mostly of selected items from the larger comprehensive ChYMH [34], with some additional items specific for screening purposes. The full interRAI ChYMH instrument is a more comprehensive standardized measure that is used to assess mental health needs more extensively. It is comprised of approximately 400 clinical elements that are used to assess psychiatric, social, environmental, and medical issues for school-age children. Both the ChYMH Screener and full ChYMH assessment tools are divided into a number of subsections, such as: demographic information; mental state indicators; substance use or excessive behaviour; harm to self and others; behaviour; cognition, communication, and development; stress, trauma, and social relationships; and education. Furthermore, both of these instruments incorporate a variety of scales and algorithms known to have strong reliability and validity [36–39]. For example, an empirical investigation assessing the inter-item reliability of several of the embedded scales, such as the Aggressive/Disruptive Behaviour Scale, Anxiety Scale, Caregiver Distress Scale, and Peer Conflict Scale, demonstrated that they had strong internal consistency with Cronbach's alpha higher than 0.70 [36].

Detailed manuals support the child/youth suite of instruments and provide coding rules for the items. The result is a valid and reliable set of information that can be used individually for case documentation and to inform program planning, as well as collectively for system reporting and secondary research purposes. All interRAI instruments and assessments are rigorously evaluated to ensure stringent psychometric properties suitable for international implementation for both adults [40–42], children and youth [38, 39, 43–46].

### Procedure

The interRAI ChYMH Screener (ChYMH-S) was administered as part of typical practice for children and youth seeking mental health services in 59 agencies across Ontario. All assessors completed a full-day training session regarding how to administer and score the ChYMH-S. The service providers that administered the ChYMH-S ranged in discipline and expertise and included psychologists, nurses, psychiatrists, speech and language therapists, child and youth workers, physiotherapists, resource teachers, developmental social service workers, and social workers. Through a semi-structured interview, either in person or over the phone, assessors gathered information from a variety of sources (i.e., family members, community members, document review, and clinical observations).

Assessment information was entered into a de-identified web-based software, password protected, encrypted, and stored on computers with no internet or USB ports to ensure confidentiality. This web-based software securely stores the data at interRAI Canada and provides each case a randomly assigned, study-specific participant number. Importantly, personal identifiers had been removed prior to the data being made available for analysis. Approval was granted through Western University’s ethics board (REB #106415) for the secondary analysis of data collected in various agencies throughout the Province of Ontario.

### Analysis

The goal was to develop an algorithm that would produce an ordinal scale of the individual’s risk of suicide and self-harm. A single ordinal item records the assessor’s perceived risk of the individual’s “danger to self” scored as 0 (minimal) to 4 (very severe or imminent). The analytic approach was to use this estimate as the dependent measure and to use multiple measures from the screener items to predict it. An important purpose of this scale will be its use with the comprehensive ChYMH assessment, which shares many, but not all, items with the ChYMH-S. Therefore, all explanatory items used in the algorithm must be available in the ChYMH assessment as well.

Importantly, in the full ChYMH, the item “danger to self” is not recorded as it is in the ChYMH-S. The algorithm development therefore uses the availability of “danger to self” from the ChYMH-S as the dependent variable in order to model or predict this in the ChYMH where it is not available. This single item reflects the clinician’s evaluation based on all available evidence as to the level of risk of the child’s danger to self.

In developing the scale, all screener records were used because it represents the flow of assessments for which to establish the relationship between predictor variables and global risk; and so this is aptly influenced by a young person who may have been screened two or more times (e.g., within both inpatient and outpatient settings). Therefore, the sample population represents the properly balanced sample of cases for which the algorithm hopes to be applied to.

Exploratory work considered modeling dichotomous proportions (e.g., percentage 2 or greater) in addition to weighted and unweighted mean values. The simple unweighted mean was ultimately chosen. The independent or explanatory variables consisted of those that were judged to be acceptable from a face validity and practical perspective. For example, while age and sex might be associated with differential self-harm risk, it is more desirable that mental health symptoms and behaviours, some of which may be age or sex related, be used instead. Also, items related to school were avoided, as not all screened children and youth are in school.

Modeling was done utilizing an interactive decision tree tool supported by the SAS Enterprise Miner package. In this interactive decision tree approach, like other tree modeling approaches, the user starts with all cases and sequentially divides them at nodes to arrive at mutually exclusive and exhaustive classifications. The SAS program allows the analyst to control, based on presented options ranked by statistical strength, which variable is chosen (for statistical and clinical reasons) and to see the resulting groups before proceeding. This allows many alternative trees to be explored. Decision trees naturally handle interactions that are common in health data, such that the influence of a given variable is specific to subsets of the data, in contrast to regression modeling which uses the average effect across all subjects. In decision tree modeling, the first split is particularly important. In addition to the top-ranking variables, forced splits (age, sex) were also considered as the first splits, but were abandoned since they offered no additional explanatory power and resulted in some fragmentation and small cell sizes in some branches. The chosen tree was subsequently tested among sex and age groups.

A design goal was to have a final scale that had a compact range: 7 groups (labels of 0 to 6, higher values with higher risk). Because of the large sample with many variables, resulting trees could have 30 or more terminal nodes, requiring them to be combined after modeling. Group assignment was done using weighted k-means clustering, resulting in 7 groups with the largest overall differences across the group means. During modeling, this resulting assignment would often result in a part of the original tree being unnecessary (over-branched), for example, a final two-way split in a branch for which both resulting nodes were assigned to the same clustered group. In these cases, the tree would be pruned at these logical points with other splitting options explored before repeating the process, until a finished parsimonious tree was designed.

Multinomial logistic regression was applied to test model fit of the dependent variable, to provide odds ratios as well as the C-statistic (area under curve). Using the full sample of 25,104 ChYMH and ChYMH-DD assessments, the RiSsK scale was calculated, and additional descriptive analyses related to diagnoses were conducted. SAS 9.4 and SAS Enterprise Miner 14.1 were used for the analysis.

## Results

**Fig. 1:**
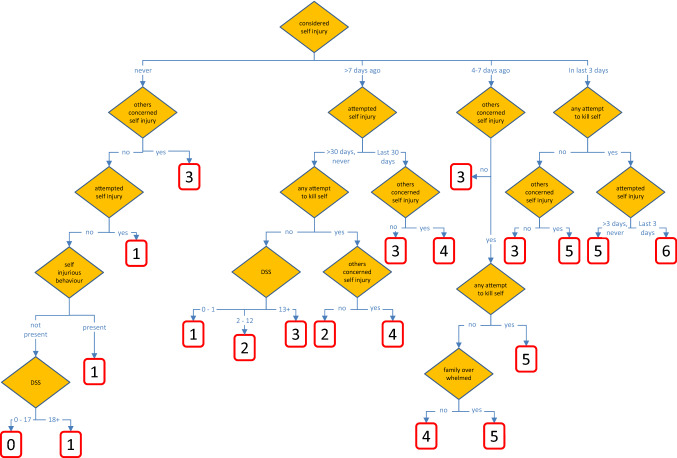
Risk of Suicide and Self-harm (RiSsK) decision tree diagram. *DSS*  depression symptoms scale

Figure 1 provides a schematic representation of the final RiSsK algorithm, which categorizes assessed children and youth into levels of risk that suggest the need for heightened concern for suicide or self-harm based on criteria from the ChYMH-S. A tree with 20 terminal nodes was selected. It uses six items from the screener assessment, plus a scale of depression symptoms (the Depression Symptoms Scale, DSS) constructed from an additional nine items. All of these 15 items exist in the same form in both the ChYMH Screener and ChYMH full assessment.

As shown in Table 1, groups were assigned from 0 (lowest) to 6 (highest) with higher scores indicating heighted risk of suicide or self-harm. The child or youth may fall into a given level via a number of pathways that represent different combinations of the criteria/risk factors. Higher risk was concentrated in a small minority of children, youth and their families [approximately 0.08% scored at the highest level (6)]. Specifically, Group 6 had 29 times the mean risk value as group 0, and 313 times the proportion rated as severe, very severe, or imminent risk. The C-statistic represents the area under the receiver operator characteristic (ROC) curve, and for the derivation dataset was 0.837. To validate the RiSsK score, data from 2117 new records were utilized. The C-statistic for the validation sample was 0.822. Table 2 presents the results for the validation sample.

**Table 1:** Derivation results of Risk of Suicide and Self-harm (RiSsK) algorithm (*N* = 60,414)

Scale label	% of sample	Mean risk	% severe, very severe, or imminent risk	Odds ratio	Low 95% confidence interval	High 95% confidence interval
0	46.3	0.08	0.1	Reference		
1	12.8	0.30	1.1	4.4	4.1	4.7
2	14.2	0.62	1.5	12.5	11.8	13.3
3	13.7	0.85	3.3	21.2	19.9	22.6
4	6.6	1.44	9.5	74.4	68.8	80.3
5	5.7	1.74	19.5	134.8	124.1	146.5
6	0.8	2.28	42.6	422.1	352.8	504.9
				c-statistic = 0.837

**Table 2:** Validation results of Risk of Suicide and Self-harm (RiSsK) algorithm (*N* = 2117)

Scale label	% of sample	Mean risk	% severe, very severe, or imminent risk	Odds ratio	Low 95% confidence interval	High 95% confidence interval
0	49.1	0.10	0.2	Reference		
1	13.0	0.34	0.7	3.9	2.7	5.5
2	14.9	0.67	1.6	11.8	8.6	16.9
3	11.8	0.82	2.8	16.0	11.5	22.1
4	6.2	1.44	10.6	61.7	41.2	92.5
5	4.2	1.73	20.2	111.0	69.1	178.2
6	0.8	2.63	50.0	683.0	253.0	> 999.99
				c-statistic = 0.822		

For the derivation sample, three different cut-points were examined by further collapsing the RiSsK score into dichotomous groups (Mild +, Moderate +, and Severe +) to determine sensitivity and specificity for identifying high risk cases. Table 3 presents sensitivity and specificity as well as positive predictive and negative predictive values, for the severe, moderate, and mild groups. As can be seen from Table 3 (severe group), a RiSsK score of 3 + provides a sensitivity of 86% and specificity of 75%. However, given the importance of sensitivity rather than specificity in relation to clinical utility, a 2 + cut-point was chosen which provided a sensitivity of 93% and a specificity of 61%. While there is likely to be more false positives with the lower threshold, a lower cut-point will reduce the likelihood that a child or youth is at high risk but is not identified.

**Table 3:** Sensitivity and specificity results for the derivation sample: mild, moderate, and severe

	RiSsK	Sensitivity (%)	Specificity (%)	PPV (%)	NPV (%)
Predict *mild or greater* risk of harm to self	1 +	90.5	64.0	54.7	93.3
	2 +	81.1	78.4	64.4	89.6
	3 +	60.1	89.4	73.1	82.3
	4 +	35.1	97.6	87.4	75.7
Predict *moderate or greater* risk of harm to self	1 +	96.2	52.8	23.9	98.9
	2 +	91.1	66.8	29.7	98.0
	3 +	77.9	81.1	38.8	96.0
	4 +	55.1	93.4	56.3	93.1
Predict *severe or greater* risk of harm to self	1 +	97.9	47.6	5.3	99.9
	2 +	93.0	60.6	6.7	99.7
	3 +	86.0	75.1	9.4	99.4
	4 +	70.6	88.7	15.9	99.0

*PPV* positive predictive value, *NPV* negative predictive value

Tables 4 and 5 present the derivation sample by age group and sex, respectively. As can be seen from Table 4, older children scored higher on the RiSsK algorithm indicating that they were at higher risk of suicide and self-harm, than younger children. Specifically, at a cut-point of 2 + for children under 7 years of age, only 10.7% (vs. 7% for a cut-point of 3 +) were classified as high on the RiSsK algorithm compared to higher scores for children aged 8–11 years (23.3% vs. 14% for a cut-point of 3 +) and those over 12 years (58.1% vs. 38.5% for a cut-point of 3 +). Additionally, girls scored higher on the RiSsK algorithm than boys (51.4% vs. 30.3% for a cut-point of 2 + and 35.1% vs. 18.2% for a cut-point of 3 +) reflecting higher risk for self-injury in the former group than the latter one.

**Table 4:** High risk for derivation results of Risk of Suicide and Self-harm (RiSsK) algorithm by age

Scale label	7 and younger		8 to 11		12 and older	
	% of sample	Odds ratio	% of sample	Odds ratio	% of sample	Odds ratio
0	66.0	Ref	59.9	Ref	34.1	Ref
1	23.4	5.6	16.8	5.3	7.8	3.6
2	3.7	16.3	9.3	15.4	19.6	9.5
3	5.1	26.6	9.2	25.8	18.3	16.1
4	0.7	67.7	2.3	101.0	10.3	55.9
5	1.1	140.3	2.2	155.2	8.7	104.1
6	0.1	872.4	0.3	436.9	1.2	329.1
c-statistic		0.789		0.831		0.815

**Table 5:** High risk for derivation results of Risk of Suicide and Self-harm (RiSsK) algorithm by sex

Scale label	Males		Females	
	% of sample	Odds ratio	% of sample	Odds ratio
0	54.0	Ref	38.7	Ref
1	15.7	4.2	9.9	4.6
2	12.1	11.1	16.3	14.7
3	10.7	19.1	16.6	24.4
4	3.8	70.3	9.3	84.8
5	3.3	113.8	8.1	160.4
6	0.4	429.3	1.1	472.7
c-statistic		0.819		0.841

To examine diagnoses related to the RiSsK algorithm, data from the full ChYMH and ChYMH-DD assessments were utilized within the same time period as the derivation sample, comparing the diagnosis recorded as most important as well as if a diagnosis was prevalent at all. Table 6 (prevalence of suicide and self-harm by diagnosis) presents the proportions that scored with higher values of the RiSsK algorithm, among those with these diagnoses. The diagnoses with higher RiSsK values varies somewhat depending on the cut-point, here either 2 + or 3 + . However, the leading three diagnoses associated with higher RiSsK algorithm scores included Mood, Adjustment and Eating Disorders.

**Table 6:** High risk for derivation results of Risk of Suicide and Self-harm (RiSsK) algorithm by DSM diagnosis

DSM-IV^a^N = 25,104 full ChYMH or ChYMH-DD	RiSsK 2 +		RiSsK 3 +	
	Most important dx (%)	Any importance (%)	Most important dx (%)	Any importance (%)
Reactive attachment	48.5	54.8	32.4	35.6
Attention deficit hyperactivity	34.0	39.9	16.7	21.1
Disruptive behaviour	43.9	44.1	23.4	23.3
Learning or communication	34.8	39.5	19.9	21.4
Autism spectrum	35.6	38.2	19.3	20.9
Substance related	53.5	57.7	30.3	30.9
Schizophrenia/psychotic	52.7	59.1	28.0	36.5
Mood	76.0	70.4	48.7	42.9
Anxiety	49.3	51.6	27.5	29.6
Eating	64.2	67.1	43.4	47.4
Sleep	42.0	50.1	26.1	30.1
Adjustment	73.8	66.6	53.7	43.3

^a^Among assessments with this diagnosis, this is the proportion reaching this RiSsK threshold

## Discussion

High risk for suicidality and self-harm was predicted by a number of different contributors. Several of the predictor variables were related to a prior history of suicidal thoughts and behaviours, as well as mental health issues and family factors. Children and adolescents who have considered or attempted self-injury, attempted to kill him or herself, or have previously engaged in any type of self-injurious behaviour received higher scores on the RiSsK algorithm. Furthermore, higher risk for suicidality and self-harm was also associated with others being concerned about the youth partaking in self-injury. This critical link between prior ideas, attempts, or acts of suicidality and self-harm, and future risk of self-harming behaviour is well-supported by the literature. Consistent with previous research, suicidal ideation is the only factor more strongly related to attempted suicide than NSSI, after controlling for psychological and demographic factors [47]. Although the literature has made an important distinction between NSSI and SSI, these two self-harming behaviours tend to co-occur, such that nonsuicidal self-injury (e.g., cutting, burning, scratching) has consistently been found to be an important risk factor for attempted suicide among adolescents [18, 48, 49]. Past research also supports our finding that attempted suicide is an important risk factor for future suicidality and self-harm; for example, one study found that 36.9% of males and 61.9% of females who completed suicide had a prior history of attempts [31]. Interestingly, adolescents who report both a history of NSSI and SSI are at increased risk for psychopathology and psychosocial impairment compared to individuals who engage in either NSSI or SSI only [17]. This suggests that individuals who engage in multiple forms of self-harm may represent a more severe clinical group and thus require more intensive resources, which is in line with the current study’s findings.

Depressive symptomology was another factor that contributed to higher scores on the RiSsK algorithm. This finding is in accordance with previous studies that have shown higher levels of depressive symptoms are associated with increased likelihood of engaging in suicidal behaviour or self-harm [12]. Furthermore, a multi-national study examining the influence of various psychosocial factors on NSSI in adolescents found that depressive symptoms was the only factor associated with increased odds of engaging in NSSI across all countries included in the study [50].

The final contributing factor to the RiSsK algorithm is the family being overwhelmed by the child or youth’s condition. There are a number of reasons the family may be feeling overwhelmed or stressed. Family members may have significant concerns about the safety of their child. Additionally, these families may not possess effective coping strategies needed to help navigate their child’s difficult situation, or the child’s condition may be compounded by other family stressors, resulting in feeling burdened or overextended. Research has shown a variety of familial factors are associated with suicidality and self-harm, such as poor family environment and low parental monitoring [51]. Interestingly, adolescents who are more likely to report a lack of family network availability have a higher likelihood of suicide attempts [52]. Families may feel overwhelmed because of the lack of outside help from family and/or friends, thus contributing to the child’s increased risk of self-harm, a finding consistent with this study. Studies have also found that low family support predicts suicidal ideation and behaviours across both gender and ethnicity [53]. It can be postulated that when a family is feeling overwhelmed by the child, family members may not be capable of providing the necessary, ongoing support the child needs, thus resulting in the increased likelihood of the child engaging in self-harming behaviours. Overall, it appears that the predictive ability of the family feeling overwhelmed within our model can be understood within the greater context of the well-documented relationship between family stressors and increased risk of self-injurious behaviours among adolescents.

The current study also examined diagnoses related to the RiSsK algorithm, and identified mood, adjustment, and eating disorders among the top diagnoses associated with higher RiSsK scores. The association between these DSM-diagnoses and higher risk for self-harm and suicidal behaviour is well-supported by the literature. For example, one of the most common mood disorders in children and youth is major depressive disorder, and as evidenced by both the results of the current study and prior research, depressive symptomology is an important independent predictor of self-injurious behaviour [13, 14].

Other research has also reported that suicidal behaviour and self-harm is very prevalent in youth with eating disorders. Koutek and colleagues found suicidal behaviour to be present in 60% of patients with an eating disorder, and self-harm in 49% [54]. Furthermore, when examining the relationship between suicidality and eating disorders across the lifespan, it has been reported that approximately 16.9% of those with anorexia nervosa have attempted suicide at some point in their lives [55].

Past literature has also found a strong relationship between adjustment disorder and suicidality. A study conducted by Gradus and colleagues found that after controlling for a number of factors including marital status, income, and history of depression, individuals with adjustment disorder had 12 times the rate of completed suicide compared to those who had not received this diagnosis [56]. Furthermore, among adolescents who were admitted to a psychiatric hospital, adjustment disorder was more common in youth hospitalized for a suicide attempt compared to those without a history of suicide attempt [57].

Overall, a number of variables predict risk for suicidality and self-harm within childhood and adolescence, including factors related to a prior history of self-injurious behaviours, mental health concerns and family stressors. Furthermore, certain DSM-diagnoses are more strongly associated with a higher RiSsK score.

### Use and Utility of RiSsK

Based on the findings, RiSsK is an empirically based decision-support tool that may be used to identify children and adolescents who exhibit symptoms that have been shown to increase one’s likelihood of engaging in self-harm and suicidal behaviour. Given that the RiSsK algorithm was found to be a strong predictor of high-risk self-injurious behaviour, service providers utilizing this decision-support algorithm will be able to make more systematic evaluations in determining whether a child or adolescent is at heightened risk of committing suicide or engaging in self-harm.

The results of the RiSsK can be obtained automatically by the assessor who has completed the interRAI ChYMH-S assessment from the software in which the algorithm is embedded. The results are intended to help support service providers in selecting appropriate resources based on the child or youth’s RiSsK score. Furthermore, the RiSsK algorithm is meant to be used in conjunction with other information obtained during the assessment process, for the purpose of assisting the clinical team in determining the level of risk of suicide or self-harm. Importantly, the RiSsK algorithm should not be used as an automated decision-making system, without any clinical judgment. Rather, the responsibility lies within the clinical team to use their professional judgment in making the decision as to whether the RiSsK score accurately reflects the child or youth’s risk of committing suicide or engaging in self-harming behaviour, when all available information is considered holistically. Finally, the child or youth, along with his or her family, should be included in the decision-making process as necessary, as each unique case requires careful consideration of that individual’s preferences, strengths, and needs.

Depending on whether the child’s RiSsK score falls within the upper or lower range will help determine subsequent care planning steps. If the child or youth’s score falls within the lower range, it is recommended that the clinical team engage in further discussion to determine whether the RiSsK level seems appropriate given all other assessment information. If the child or youth’s score falls within the upper range, it is recommended that the clinical team consider the individual to be at high risk for self-harm or suicide. The Suicidality and Purposeful Self-Harm collaborative action plan (CAP) developed by interRAI can assist clinicians in care planning for higher risk children and adolescents [58, 59]. When the young person is at high risk of self-harm, immediate safety planning is required. When the young person is at moderate risk of self-harm, clinicians can consider referral to in-patient or out-patient treatment depending on the circumstances, such as whether the child has a supportive family relationship and stable home life, or whether the child is experiencing extreme levels of uncontrollable stress. Early detection and intervention are essential components of effective prevention efforts, which signifies the importance of the RiSsK algorithm in identifying children and youth who are at a greater risk of suicide or self-harm, as it enables clinicians to intervene earlier on.

Notably, proper assessment of self-harm is associated with increased resources and costs in the mental health field. This is evidenced by a study that examined predictors of service complexity in children’s mental health, and found that suicidality risk and purposeful self-harm was an important contributor to increased resource costs [46]. Furthermore, other research has found that self-harm and suicidal ideation are associated with increased use of emergent and intensive services as a result of these behaviours being life-threatening by nature [11, 60]. Taken altogether, this finding emphasizes the need to properly assess and care plan around this critical issue, again pointing to the significant utility of the RiSsK algorithm.

RiSsK also has potential benefits beyond individualized care planning, such as providing comprehensive, standardized data across large catchment areas. Moreover, it can be implemented across multiple service sectors (e.g., schools, emergency departments, policing, child welfare, developmental services, mental health facilities, universities). Specifically, interRAI instruments utilize core items across existing assessment instruments that evaluate self-harm and suicide risk, thereby allowing an opportunity for an integrated health information system [61] (please see www.interRAI.org). This broader application is dual purpose in that it facilitates the identification of risk of suicide and self-harm across the system, and can help justify expenditures [62]. In line with other interRAI algorithms, RiSsK allows for population stratification according to level of need, which enables comparisons to be made between the performance of mental health agencies with respect to outcomes of care within the RiSsK levels [46, 62]. As a result, practice patterns can be evaluated at multiple levels (i.e. regional, organizational, national and international) [63]. Additionally, RiSsK levels at intake can be used to help examine differences in how services are utilized by level of need across various regions. The key advantage in implementation of the RiSsK algorithm would be that children and youth with higher levels of need would receive more extensive services and resources than those with lower-level need. It is important to note, however, that this does not mean children and youth scoring at the lowest level of risk are not given appropriate resources.

While this study has numerous strengths, including its relatively large sample size, it is not without its limitations. First, this study is cross-sectional in nature. As a result of this, older children have had a longer opportunity to engage in suicidal and self-harming behaviours, and so it may not be unexpected that these children obtained higher RiSsK scores compared to younger children. In the future, longitudinal data are needed to examine risk of suicidality and self-harm as the child grows and develops. Second, the findings may not be generalizable to a community-based sample because the children and youth assessed were accessing outpatient or inpatient mental health services. Additional future research should examine whether this study’s findings are consistent when participants comprise of a community sample.

## Summary

Suicide is a major public health concern as it is the second leading cause of death among Canadian youth [2]. Although a number of risk factors have been previously identified, there continues to be a critical need for the development of appropriate decision-support tools to identify young persons who are at highest risk for suicide and self-harm. In this study, analyses were conducted using the interRAI ChYMH-S data collected within the province of Ontario to develop a decision-support algorithm to identify children and youth at risk for suicide or self-harm. The primary sample for the derivation of the algorithm was 60,414 records obtained from participants aged 4–18 years who had completed the ChYMH-S assessment. The algorithm was subsequently validated with additional data from 59 agencies. The independent or predictor variables that contributed to the RiSsK algorithm included having considered or attempted self-injury, attempted to kill him or herself, have previously engaged in any type of self-injurious behaviour, others being concerned about the youth partaking in self-injury, along with depressive symptomology and the family being overwhelmed by the child or youth’s condition. Through the derivation and validation efforts, the RiSsK algorithm was found to be a strong predictor of suicide and self-harm. Therefore, the RiSsK algorithm provides a psychometrically sound decision-support tool that may be used to identify children and adolescents at heightened risk of engaging in suicide or self-harm. The main goal for the development and implementation of the RiSsK algorithm is to prevent future self-harming behaviours or suicidal acts through the earlier detection, and subsequent intervention, of children and youth at greatest risk.
